# VacA promotes CagA accumulation in gastric epithelial cells during *Helicobacter pylori* infection

**DOI:** 10.1038/s41598-018-37095-4

**Published:** 2019-01-10

**Authors:** Majd Abdullah, Laura K. Greenfield, Dana Bronte-Tinkew, Mariana I. Capurro, David Rizzuti, Nicola L. Jones

**Affiliations:** 1 Departments of Paediatrics and Physiology, University of Toronto, Toronto, Ontario Canada; 2 Cell Biology Program, Research Institute, Hospital for Sick Children, Toronto, Ontario Canada

## Abstract

*Helicobacter pylori* (*H*. *pylori*) is the causative agent of gastric cancer, making it the only bacterium to be recognized as a Class I carcinogen by the World Health Organization. The virulence factor cytotoxin associated gene A (CagA) is a known oncoprotein that contributes to the development of gastric cancer. The other major virulence factor vacuolating cytotoxin A (VacA), disrupts endolysosomal vesicular trafficking and impairs the autophagy pathway. Studies indicate that there is a functional interplay between these virulence factors by unknown mechanisms. We show that in the absence of VacA, both host-cell autophagy and the proteasome degrade CagA during infection with *H*. *pylori*. In the presence of VacA, CagA accumulates in gastric epithelial cells. However, VacA does not affect proteasome function during infection with *H*. *pylori* suggesting that VacA−disrupted autophagy is the predominant means by which CagA accumulates. Our studies support a model where in the presence of VacA, CagA accumulates in dysfunctional autophagosomes providing a possible explanation for the functional interplay of VacA and CagA.

## Introduction

*Helicobacter pylori* (*H*. *pylori*) is a gram-negative bacterium that colonizes the gastric mucosa in over 50% of the population worldwide causing gastritis in all infected individuals, peptic ulcers in 10–20% and gastric cancer in 1–2% of those infected^1,2^. The World Health Organization has classified *H*. *pylori* as a Class I carcinogen for gastric cancer development^3^. Gastric cancer accounts for approximately 754,000 deaths per year and is the 4^th^ leading cause of cancer-related deaths worldwide^4^. The presence of two main virulence factors, the cytotoxin associated gene A (CagA) and the vacuolating cytotoxin A (VacA), are associated with an increased risk of developing gastric cancer^5,6^.

CagA is a 120–145 kDa oncoprotein injected into host cells by a type IV secretion system^7^. Once internalized, CagA is tethered to the inner leaflet of the plasma membrane where it can be phosphorylated on tyrosine residues in EPIYA motifs by Src and Abl kinases^8^. The oncogenic capability of CagA has been directly demonstrated through the use of transgenic mice and zebrafish models that developed gastrointestinal tumors^9,10^. Further increasing our understanding of the regulation of CagA during host-pathogen interactions should advance the development of novel preventative and therapeutic approaches to combat carcinogenesis.

Cellular proteins are targeted for degradation by the ubiquitin-proteasome system or the autophagy pathway^11^. Tsugawa *et al*. have demonstrated that CagA is degraded by autophagy^12^. Importantly, work from our laboratory and others has shown that VacA can modulate the autophagy pathway^13–16^.

VacA is a secreted pore-forming cytotoxin that upon acute exposure stimulates the autophagy pathway in cells^12–14^. However, upon prolonged exposure, VacA disrupts lysosomal trafficking, resulting in the accumulation of dysfunctional autophagosomes that lack cathepsin D and the formation of large intracellular vacuoles that promote the intracellular survival of *H*. *pylori*^15^. All strains of *H*. *pylori* harbor the *vacA gene*, suggesting that VacA plays a crucial role in the colonization and persistence of *H*. *pylori* within the gastric mucosa^17^. Previous studies have demonstrated antagonistic interactions between VacA and CagA^18–20^. This antagonism is evident morphologically where isogenic *cagA* mutant strains induced greater vacuolation, while isogenic *vacA* mutant strains have more pronounced hummingbird phenotype, a hallmark of CagA intoxication, compared to wild-type strains^21^. In fact, CagA has been shown to reduce the entry of VacA into host cells^22^. Although the exact mechanisms underlying the functional antagonism between the two virulence factors remains unclear, studies have shown that effects on various intracellular pathways, including NFAT, apoptosis, and MAP kinase have been proposed to play a role^18–20^.

Emerging evidence in the past decade has demonstrated considerable “cross-talk” between the ubiquitin-proteasome system and the autophagy pathway^23^. During proteasome inhibition/dysfunction, autophagy can serve as a compensatory mechanism to clear ubiquitinated substrates^24,25^. Conversely, autophagy inhibition/dysfunction is not compensated by enhanced proteasome activation^26,27^. In fact, prolonged disruption of autophagy has been shown to hinder proteasome degradation and leads to an accumulation of proteasome substrates^28^. Therefore, we determined the role of autophagy and the proteasome in the regulation of CagA levels. Furthermore, since VacA results in accumulation of disrupted autophagosomes, we characterized the impact of VacA on autophagy, the proteasome and CagA levels.

## Results

### Both autophagy and the proteasome regulate intracellular CagA

**Figure 1:**
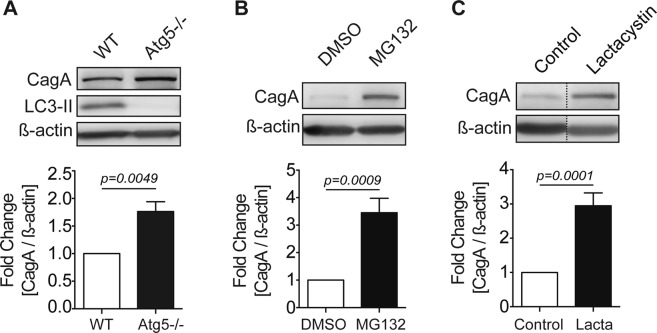
Autophagy and the proteasome regulate CagA stability. (**A**) Wild-type (WT) and autophagy-deficient (Atg5−/−) MEFs were infected with a CagA+ *vacA*− isogenic mutant strain (MOI 100) for 8 hours using a gentamycin protection assay. The autophagy marker LC3-II was used to confirm that Atg5−/− MEFs are autophagy-deficient. CagA protein levels were measured by Western blotting using β-actin as loading control. Graph shows fold change of CagA normalized to β-actin relative to WT MEFs (mean + SEM; n = 4). (**B**) AGS cells were infected with a CagA+ *vacA*− isogenic mutant strain (MOI 50) for 19 hours using a gentamycin protection assay. MG132 (5 μM) was added during the 14-hour low-dose gentamycin incubation. DMSO was used as a vehicle control. CagA protein levels were measured by Western blotting using β-actin as loading control. Graph shows fold change of CagA normalized to β-actin relative to vehicle control (mean + SEM; n = 6). **(C)** AGS cells were infected with a CagA+ *vacA*− isogenic mutant strain (MOI 50) for 24 hours using a gentamycin protection assay. Lactacystin (10 μM) was added during the 19-hour low-dose gentamycin incubation. Water was used as a vehicle control. CagA protein levels were measured by Western blotting using β-actin as loading control. Graph shows fold change of CagA normalized to β-actin relative to vehicle control (mean + SEM; n = 5). Relevant gel bands were cropped from the original blots. Dotted line indicates slicing of two regions together from the same blot. Statistical analysis was performed using Student’s t-test.

Cellular proteins can be degraded by autophagy or selectively targeted for degradation by the ubiquitin-proteasome system^11^. Therefore, we assessed the role of autophagy in regulating CagA by infecting autophagy-deficient cells with *H*. *pylori*. To eliminate any effects of VacA, which we have shown disrupts autophagosome maturation^15^, we infected autophagy-deficient (Atg5−/−) Mouse Embryonic Fibroblasts (MEFs) with a CagA+ *vacA-* isogenic mutant strain of *H*. *pylori* for 8 hours using a gentamycin protection assay and measured intracellular CagA levels by Western Blot. An increase in CagA was detected in infected Atg5−/− MEFs in comparison to wild-type (WT) cells (Fig. 1A). Parallel viability assays were performed to quantify the number of intracellular bacteria and determine if the observed increase in CagA could be due to an increase in bacterial survival. We normalized the levels of CagA to the level of intracellular bacteria as determined by colony forming units (CFU). After controlling for intracellular survival, the increase in CagA levels in Atg5−/− MEFs persisted (Fig. S1A). To further validate our findings, we used siRNA to knockdown Atg12 in gastric epithelial (AGS) cells and infected cells with a CagA+ *vacA*− isogenic mutant strain of *H*. *pylori* (Fig. S2A). Similar to the findings with the Atg5−/− MEFs, an increase in CagA was detected in AGS cells with siRNA knockdown of Atg12, in comparison with control cells.

To determine if the proteasome could also regulate CagA levels, we treated AGS cells with proteasome inhibitors and measured intracellular CagA levels. AGS cells infected with a CagA+ *vacA*− isogenic mutant strain of *H*. *pylori* and treated with the proteasome inhibitor MG132 demonstrated an increase in CagA compared to vehicle control (Fig. 1B). We confirmed these findings using a different proteasome inhibitor, lactacystin, which showed a similar increase in CagA levels (Fig. 1C). We performed parallel viability assays to determine if the proteasome influenced intracellular bacterial survival. There was no significant difference in the number of intracellular bacteria in cells treated with proteasome inhibitors compared to control (Fig. S1B-C). These results indicate that intracellular CagA levels are regulated by both autophagy and the proteasome.

### VacA promotes CagA accumulation during *H. pylori* infection

**Figure 2:**
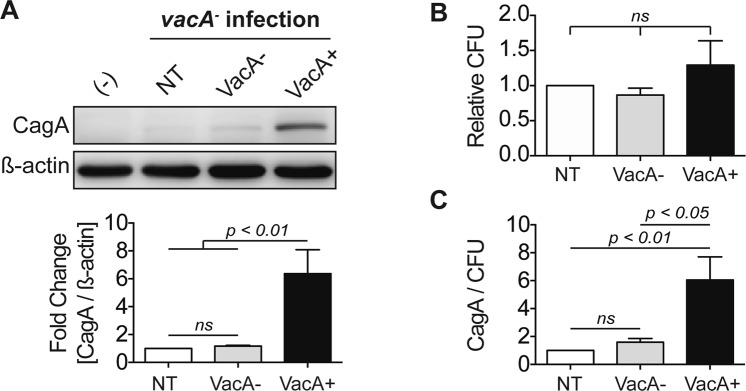
VacA promotes CagA accumulation. (**A**) AGS cells were infected with a CagA+ *vacA*− isogenic mutant strain and co-cultured in the presence or absence of VacA− or VacA + CCMS for 24 hours using a gentamycin protection assay to assess CagA levels. NT denotes infection with a CagA+ *vacA*− isogenic mutant strain alone and (-) denotes uninfected control cells. CagA protein levels were measured by Western blotting using β-actin as loading control. Relevant gel bands were cropped from the original blots. Graph shows fold change of CagA normalized to β-actin relative to NT (mean + SEM; n = 5). **(B)** Bacterial viability assays were performed in parallel to calculate colony forming units (CFU). Graph shows the average CFU relative to NT (mean + SEM; n = 5). **(C)** Graph shows CagA protein level normalized to β-actin and CFU from each corresponding experiment (mean + SEM; n = 5). Statistical analysis was performed using ANOVA with Tukey’s post-hoc test.

Since VacA disrupts both autophagy and lysosomal degradation within the cell^15^, we further investigated whether VacA can alter intracellular CagA levels during *H*. *pylori* infection. AGS cells were infected with a CagA+ *vacA*− isogenic mutant strain of *H*. *pylori* and co-cultured with or without conditioned culture media supernatant (CCMS) obtained from the wild-type VacA+ strain or the vacA− isogenic mutant strain of *H. pylori* for up to 24 hours. We confirmed that VacA disrupts autophagic degradation by assessing LC3-II and p62 accumulation (Fig. S3A-B). Following a gentamycin protection assay, cell lysates were tested for intracellular CagA levels. Incubation with VacA+ CCMS significantly increased CagA levels compared to untreated or VacA− CCMS treated cells infected with *H*. *pylori* for 24 hours (Fig. 2A). We have shown previously that VacA promotes intracellular survival of *H*. *pylori* during chronic infection^13^. Therefore, to determine if the observed increase in CagA in the presence of VacA could be due to an increase in bacterial survival, we normalized the levels of CagA to the level of intracellular bacteria as determined by CFUs (Fig. 2B). After controlling for intracellular survival, the increase in CagA levels in the presence of VacA persisted. This data suggests that VacA promotes an accumulation of CagA, independent of bacterial survival. We next attempted to assess the subcellular localization of CagA in the presence of VacA+ or VacA− CCMS using AGS cells transfected with CagA-GFP. In control cells, CagA was mainly localized to the cell membrane as expected. In transfected cells treated with either VacA+ or VacA− CCMS, CagA-GFP was redistributed to puncta within the cell (Fig. S2B). There appeared to be an increase in co-localization of the GFP puncta with LC3 in VacA+ CCMS-treated cells in comparison with VacA− CCMS-treated cells.

### Effect of VacA on the proteasome

**Figure 3:**
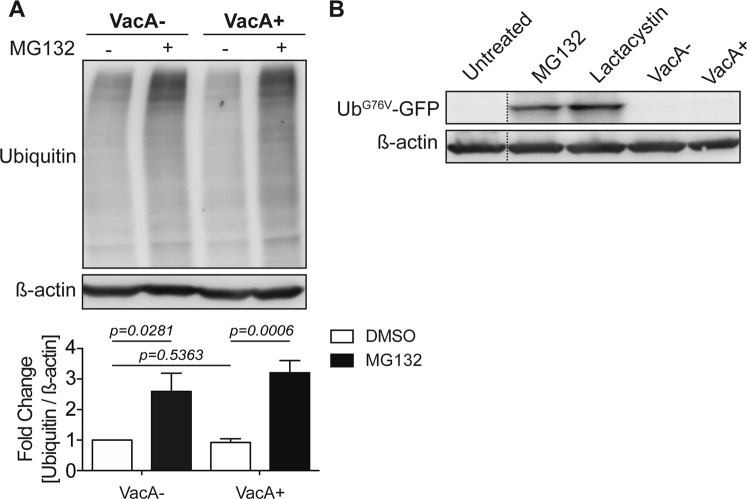
VacA disrupts autophagy but does not impair proteasome degradation. (**A**) AGS cells were infected with a CagA+ *vacA* isogenic mutant strain and co-cultured in the presence or absence of VacA− or VacA+ CCMS for 19 hours using a gentamycin protection assay. MG132 (5 μM) was added during the 14-hour low-dose gentamycin incubation. DMSO was used as a vehicle control. Ubiquitin protein levels were measured by Western blotting using β-actin as loading control. Graph shows fold change of ubiquitin normalized to β-actin relative to vehicle control (mean + SEM; n = 5). (**B**) Ub^G76V^-GFP HeLa cells were treated with 10 μM of MG132 or Lactacystin for 4 and 8 hours respectively, or VacA+ or VacA− CCMS for 32 hours. Ub^G76V^-GFP protein levels were measured by Western blotting using β-actin as loading control (mean + SEM; n = 3). Relevant gel bands were cropped from the original blots. Dotted line indicates slicing of two regions together from the same blot. Statistical analysis was performed using Student’s t-test.

Since dysregulation of autophagy has been shown to impair proteasome degradation^26–28^, we next determined if VacA, which disrupts autophagosome maturation, could alter proteasome function. We first explored whether VacA impairs proteasomal degradation by quantifying the total amount of ubiquitinated proteins during *H*. *pylori* infection in AGS cells treated with VacA+ or VacA− CCMS. There was a significant increase in the amount of ubiquitinated proteins in cells treated with MG132 (Fig. 3A). In contrast, we did not observe a significant change in ubiquitinated proteins in VacA+ treated cells in comparison with VacA− treated cells, suggesting that VacA does not alter proteasome function. To directly assess the effects of VacA on the proteasome, we used the ubiquitin-proteasome pathway activity reporter Ub^G76V^-GFP, which is degraded by the proteasome and rapidly accumulates if proteasome degradation is impaired^29^. We employed HeLa cells stably expressing the Ub^G76V^-GFP reporter to compare levels of Ub^G76V^-GFP in cells treated cells with VacA+ or VacA− CCMS. We confirmed that VacA impairs autophagic degradation in these Hela cells (Fig. S3C-D). As shown in Fig. 3B, there was minimal Ub^G76V^-GFP detected in cells treated with VacA− CCMS. Similarly, VacA+ CCMS treatment did not result in increased Ub^G76V^-GFP. As expected, an increase in Ub^G76V^-GFP was detected in cells treated with MG132 or lactacystin. Taken together these findings suggest that VacA does not impact proteasome function.

**Figure 4:**
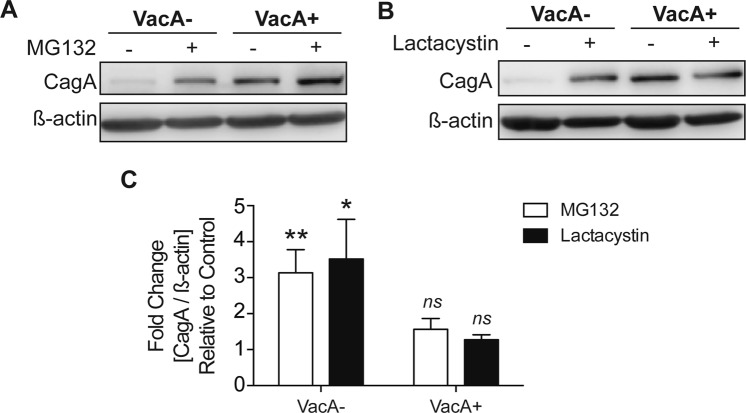
Proteasome degradation of CagA is influenced by VacA. AGS cells were infected with a CagA+ *vacA*− isogenic mutant strain in the presence of VacA− or VacA+ CCMS for (**A**) 19 hours or (**B**) 24 hours using a gentamycin protection assay and incubated with **(A)** MG132 (5 μM) or (**B**) Lactacystin (10 μM) during the 14-hour or 19-hour low-dose gentamycin incubation, respectively. DMSO and water was used as a vehicle control. CagA protein levels were measured by Western blotting using β-actin as loading control. Relevant gel bands were cropped from the original blots. (**C**) Graph shows fold change of CagA normalized to β-actin relative to vehicle control in cells treated with MG132 or Lactacystin (mean + SEM; n = 6). Statistical analysis was performed using Student’s t-test (*p < *0*.*05*, **p < *0*.*01*, *ns* = *not significant*).

In contrast to complete inhibition of autophagosome formation seen in Atg5−/− MEFs, VacA disrupts autophagosome maturation, resulting in the accumulation of dysfunctional autophagosomes. We examined the effect of proteasome inhibition in the presence of VacA. As shown in Fig. 4A–C, in the absence of VacA, proteasome inhibition promotes accumulation of CagA. However, in the presence of VacA, proteasome inhibition does not result in further increases in CagA (Fig. 4C). These findings suggest that in the presence of VacA, the functional proteasomes are not able to target CagA for degradation.

### Effects of VacA on phosphorylated CagA

**Figure 5:**
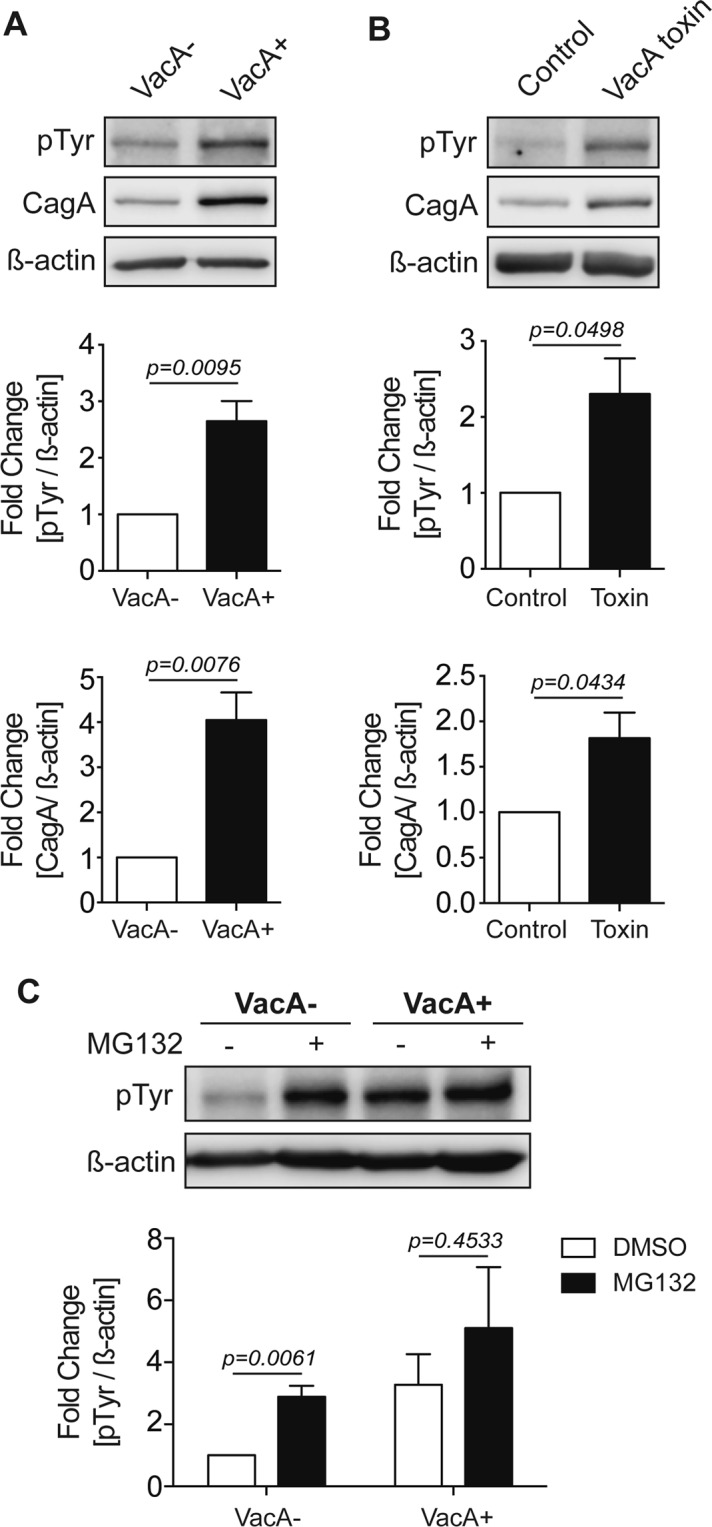
VacA promotes the accumulation of phosphorylated CagA. AGS cells were infected with a CagA+ *vacA*− isogenic mutant strain in the presence of **(A)** VacA− or VacA+ CCMS and (**B**) untreated or treated with purified VacA toxin for 20 hours using a gentamycin protection assay. Phosphorylated CagA (pTyr) and total CagA levels were measured by Western blotting using β-actin as a loading control. (**C**) AGS cells were infected with a CagA+ *vacA*− isogenic mutant strain in the presence of VacA− or VacA+ CCMS for 19 hours using a gentamycin protection assay and incubated with DMSO or MG132 (5 μM). Phosphorylated CagA levels were measured by Western blotting using β-actin as loading control. Graphs show fold change of pTyr or CagA normalized to β-actin (mean + SEM; n = 3). Statistical analysis was performed using Student’s t-test.

CagA alters specific host cell signaling pathways in both a tyrosine phosphorylation dependent and independent manner^30–36^. Furthermore, only intracellular CagA can be tyrosine phosphorylated by host cell kinases, therefore changes in phosphorylated CagA specifically reflect changes in intracellular CagA. We next determined how VacA affects the levels of tyrosine phosphorylated CagA during *H*. *pylori* infection (Fig. 5). AGS cells treated with VacA+ CCMS had a significant increase in phosphorylated CagA (Fig. 5A). Next, to confirm that these changes were specific for VacA, we treated cells with purified VacA toxin. As shown in Fig. 5B, VacA is necessary and sufficient to promote CagA and phosphorylated CagA accumulation in AGS cells infected with a CagA+ vacA− isogenic mutant strain of *H*. *pylori*.

Next, we assessed the effect of proteasome inhibition on phosphorylated CagA in the presence or absence of VacA. As shown in Fig. 5C, there was a significant increase in the amount of phosphorylated CagA in VacA− CCMS treated cells in the presence of MG132, suggesting that the proteasome targets both phosphorylated and non-phosphorylated CagA for degradation. Addition of the proteasome inhibitor to VacA+ CCMS treated cells did not result in a further increase in phosphorylated CagA levels, which is consistent with the findings assessing total CagA levels under the same conditions.

## Discussion

*H*. *pylori* is one of the most successful human pathogens due to its ability to avoid degradation and maintain a chronic infective state without killing its host. This success is mediated in part by the interplay of two key virulence factors, CagA and VacA. In this study, we show that in addition to degradation by the autophagy pathway, CagA is degraded by the proteasome. Furthermore, in the presence of VacA there is an accumulation of both total and phosphorylated CagA. However, VacA does not affect the proteasome suggesting that in the presence of VacA, oncogenic CagA accumulation is likely mediated by VacA−disrupted autophagy thereby demonstrating a complex interplay between CagA and VacA.

Previous studies have shown that CagA is a relatively short-lived protein that is rapidly degraded^37^ and that this degradation is mediated by autophagy as shown by the use of autophagy inhibitors^12^. We validated these results using Atg5−/− MEFs and Atg12 siRNA knockdown of AGS cells to show that CagA is degraded by autophagy. Parallel viability assays demonstrate that the accumulation in CagA in Atg5−/− MEFs was not due to increased bacterial survival. These findings are consistent with those of Tsugawa *et al*. who demonstrated that CagA specifically accumulates in CD44v9-expressing cancer stem-like cells which have suppressed autophagy^12^. In contrast to their study, which found that VacA exposure increased degradation of CagA, we found that VacA−disrupted autophagy promotes an accumulation of CagA. We had previously shown that acute exposure to VacA enhances autophagy while prolonged exposure to VacA disrupts autophagy^14,15^. We suggest that differences in methodology may explain this apparent discrepancy. Of interest, a recent study by Li *et al*., demonstrated that CagA could inhibit autophagy^38^. We did not assess the effect of CagA on autophagy in the present study.

In addition to autophagic degradation of CagA, our results show for the first time that the proteasome is also involved in the regulation of CagA. We found that in the presence of VacA, the degradation of CagA mediated by the proteasome is prevented. Since VacA does not impair proteasome function, we propose that CagA may accumulate in defective autophagosomes, where it is inaccessible to the proteasome. We attempted to determine the subcellular location of CagA in the presence of VacA by using cells transfected with CagA-GFP. We found that culture supernatants from both VacA+ and VacA− *H*. *pylori* caused a redistribution of CagA from the cell membrane to puncta within the cell. In addition, in cells treated with VacA+ CCMS, there appeared to be more colocalization with LC3+ puncta indicative of autophagosomes. However, this data should be interpreted with caution as transfection of CagA may not accurately reflect delivery of CagA during infection. Thus, additional studies will be required to delineate the specific subcellular location of CagA in the presence of VacA during *H*. *pylori* infection.

In summary, our studies support a model whereby VacA-mediated disruption of autophagy promotes CagA accumulation in cells. We suggest that CagA may accumulate in dysfunctional autophagosomes that remain inaccessible to the proteasome and limit potential downstream signaling of CagA. Thus, we provide a potential mechanism explaining the functional antagonism of CagA in the presence of VacA.

## Materials and Methods

### Antibodies

The following antibodies were used for Western blotting: anti-β-actin (A4700; 1:5000; Sigma-Aldrich, Oakville, Canada), anti-LC3B (NB600-1384; 1:1000 for WB; 1:200 for IF; Novus Biologicals; Oakville, Canada), anti-p62 (610832; 1:1000; BD Transduction; Mississauga, Canada), anti-pTyr (4G10; 1:500; EMD Millipore, Etobicoke, Canada), anti-CagA (b-300; 1:500; Santa Cruz, Dallas, TX), anti-Ubiquitin (P4D1; 1:500; Santa Cruz, Dallas, TX), anti-Atg12 (2010; 1:500; Cell Signaling, Whitby, Canada) goat anti-mouse IgG (115-035-003; 1:2500; Jackson ImmunoResearch, West Grove, PA), and goat anti-rabbit IgG (115-035-144; 1:2500; Jackson ImmunoResearch, West Grove, PA).

### Pharmacological inhibitors

MG132 (5 μM; Sigma-Aldrich, Oakville, Canada) was dissolved in DMSO and Lactacystin (10 μM; Enzo Life Sciences, Farmingdale, NY) was dissolved in milli-Q water. Cells were incubated with MG132 for 14 hours or Lactacystin for 19 hours during the low-dose gentamycin treatment in order to inhibit the 20 S proteasome subunit. Control cells were treated with DMSO or milli-Q water, respectively.

### Bacterial culture and growth conditions

*Helicobacter pylori* strain 60190 was purchased from American Type Culture Collection (49503, *cagA*+ , *cagE*+ , *vacA*+ ; Rockville, MD). Isogenic *vacA* and *cagA* mutants were provided by Dr. Richard Peek (Vanderbilt University School of Medicine, Nashville, TN) and were constructed as described^39,40^. *H*. *pylori* was grown on Columbia agar plates supplemented with 5% sheep blood (Oxoid Microbiology Products, Thermo Scientific, Napean, Canada) for 48–72 hours at 37 °C in microaerophilic conditions (5% oxygen, 10% carbon dioxide). *H*. *pylori* was then transferred to Brucella broth (Sigma-Aldrich, Oakville, Canada) supplemented with heat-inactivated Fetal Bovine Serum (FBS; Wisent, St. Bruno, Canada) and grown for 18–24 hours at 37 °C in microaerophilic conditions. *H*. *pylori* was then assessed by observing helical morphology as well as viability through active mobility using a light microscope. The concentration of bacteria was determined by measuring the optical density at 600 nm (OD 600), where 1 OD is equivalent to 2 × 10^8^ bacteria/mL.

### Preparation of *H. pylori* culture supernatants and purified VacA toxin

For cell treatment with VacA toxin, wild-type and *vacA* isogenic mutant *H*. *pylori* culture supernatants (OD = 1.0) were filtered through a 0.22-μm-cutoff membrane filter and concentrated 10 times using a 30-kDa-cutoff Amicon Ultra centrifugal filter (Millipore, Billerica, MA). VacA CCMS was utilized at 10% of the final concentration. Purified VacA toxin (kindly provided by Dr. Steven Blanke) was diluted 1:100 and activated by incubation in acidified Ham’s F-12K culture media, pH 2, for 30 minutes at 37 °C. The media was then neutralized to pH 7 and supplemented with 10% FBS. For cell intoxication, AGS cells were incubated in the presence of activated purified VacA toxin (35 nM) for 20 hours.

### Cell culture

AGS cells were purchased from American Type Culture Collection (Rockville, MD). AGS cells were grown in Ham’s F-12K media supplemented with 10% heat-inactivated FBS (Wisent, St. Bruno, Canada) and grown at 37 °C in a humidified chamber (5% CO_2_). Wild-type and *Atg5* deficient (*Atg5*−/−) mouse embryonic fibroblasts (MEFs) were kindly provided by Dr. John Brumell (The Hospital for Sick Children, Toronto, Canada) and grown in Dulbecco’s Modified Eagle’s Media (DMEM; Wiscent, St. Bruno, Canada) supplemented with 10% FBS. HeLa cells stably expressing the Ub^G76V^-GFP reporter was kindly provided by Dr. Nico Dantuma (Karolinksa Institutet, Stockholm, Sweden) and grown in DMEM containing 10% FBS, 0.5 mg/mL G418 (Sigma-Aldrich, Oakville, Canada), 2 mM L-glutamine, and 1% penicillin-streptomycin.

### Cell invasion and intoxication assays

For *H*. *pylori* infection, bacteria were centrifuged at 2,500 g for 5 minutes and resuspended in the appropriate cell culture media. Cell invasion was performed at a multiplicity of infection (MOI) of 50 on 70–90% confluent cells AGS cells, and an MOI of 100 on 70–90% confluent MEFs. Infected AGS cells were co-cultured with or without VacA− or VacA+ CCMS. For the gentamycin protection assays, cells were infected for 4 hours and then washed 3 times with PBS to remove non-adhered bacteria. Cells were then incubated in cell culture media containing 100 μg/mL gentamycin (Wisent, St. Bruno, Canada) for 1 hour to kill non-internalized bacteria, after which gentamycin was reduced to 10 μg/mL to prevent the growth of extracellular bacteria. VacA− or VacA+ CCMS along with pharmacological inhibitors were co-cultured with the 10 μg/mL of gentamycin for the remaining duration of the experiment.

### Viability Assays

Experiments were performed in duplicate; one set was used to prepare lysates for Western blotting and the other to measure bacterial viability. Bacterial viability assays were carried out as previously described^41^. Briefly, at the end of the invasion assays, cells were washed 3 times with PBS and lysed in a 1% saponin (Sigma-Aldrich Oakville, Canada) solution prepared in serum-free cell culture media for 8–10 minutes. Serial dilutions were prepared in serum-free cell culture media and 50 μL of each dilution was pipetted onto Columbia agar plates supplemented with 5% sheep blood in triplicate. Plates were placed at 37 °C in microaerophilic conditions for 72–96 hours before enumerating colony forming units (CFU). The average CFU value from triplicate plates was used for statistical analysis.

### Immunoblotting

Cells were lysed, as previously described^42^, and separated using SDS-PAGE followed by transfer to nitrocellulose membranes (Pall Corporation, Port Washington, NY). Membranes were probed at 4 °C overnight with the appropriate primary antibodies followed by incubation with the corresponding horseradish peroxidase-conjugated secondary antibodies for 1 hour at room temperature. Bands were visualized by chemiluminescence (Western blotting luminol reagent; Santa Cruz, Dallas, TX) using Licor Odyssey Fc imaging system.

### Statistics

Densitometry analysis was performed using Image Studio software (Licor, Lincoln, NE). Densities of proteins bands were measured and expressed as a ratio of protein of interest over the loading control (i.e. CagA/Actin). For graphical representation, the ratio of each treatment was expressed as the fold-change relative to the control. Statistical significance between treatment groups was calculated using GraphPad Prism 6.0c (GraphPad Inc., La Jolla, CA). P values less than 0.05 were deemed statistically significant.

## Supplementary information

Supplementary Dataset 1

## Data Availability

All data and constructs are available upon request.
