# Supplementary material for: VacA promotes CagA accumulation in gastric epithelial cells during Helicobacter pylori infection
Source: Sci Rep. 2019 Jan 10;9:38. doi: 10.1038/s41598-018-37095-4 (PMC6328614; doi:10.1038/s41598-018-37095-4)
Supplement: Supplementary file 1 — Supplementary Dataset 1 [file 41598_2018_37095_MOESM1_ESM.pdf]

SUPPLEMENTAL INFORMATION

MANUSCRIPT TITLE: VacA promotes CagA accumulation in gastric epithelial cells during *Helicobacter pylori* infection

Majd Abdullah[1,2], Laura K. Greenfield[1,2], Dana Bronte-Tinkew[1,2], Mariana I. Capurro[1,2], David Rizzuti[1,2], *Nicola L. Jones[1,2]

[1]Departments of Paediatrics and Physiology, University of Toronto, [2]Cell Biology Program, Research Institute, Hospital for Sick Children, Toronto, Ontario, Canada

METHODS

siRNA-mediated gene silencing

Autophagy was inhibited by transfecting AGS cells with a 21-nt siRNA directed to the human autophagic protein Atg12 (NM_004707). A sense/antisense siRNA pair targeting nucleotides 515 to 533 of Atg12 sequence, GUGGGCAGUAGAGCGAACAUdT, was purchased from Dharmacon Research Inc. Chicago. Non-targeting siRNA #1 (D-001210-01-20) was purchased from Dharmacon Research Inc. and used as a control. siRNA was delivered into AGS cells utilizing RNAiMAX (Thermo Scientific, Napean, Canada). AGS cells that were 50% confluent were treated for 24 hours with 200 pmol of siRNA and 5 µl of RNAiMAX per well of a 6-well culture plate. AGS cells were trypsinized and seeded onto 12-well plates and left to adhere for 24 hours. AGS cells were then infected with a CagA+ *vacA*- isogenic mutant strain for 24 hours before being assessed for inhibition by immunoblotting.

Transfection

The CagA-GFP construct was kindly provided by Dr. Hitomi Mimuro and constructed as described[1]. Briefly, the *cagA* gene of *H. pylori* NCTC11637 was inserted into pEGFP-C1 (Clontech) for expression of green fluorscent protein (EGFP)-tagged CagA. CagA-GFP was transfected using FuGENE HD transfection reagent (Promega, Madison, Wisconsin, USA) using a ratio of 3:1 (FuGENE:DNA) in AGS cells. After overnight transfection, AGS cells were treated with VacA+ or VacA- CCMS for 24 hours and subsequently underwent immunofluorescence staining.

Immunofluorescence

Prior to immunofluorescence labeling, cells were washed 3 times with PBS and fixed in 4% paraformaldehyde (Electron Microscopy Sciences, Hatfield, Pennsylvania, USA) for 30 minutes. After fixation, cells were permeabilized in ice-cold methanol for 15 minutes and blocked in 1% bovine serum albumin (BSA) in PBS for 1 hour. LC3 was stained overnight using a rabbit anti-LC3 antibody (1:200) in 5% BSA made in PBS. Cells were then incubated with Alexa Fluor 568 anti-rabbit conjugated antibodies in 1% BSA in PBS. Slides were mounted using DAKO Mounting medium and imaged using a Quorum Spinning Disk Confocal Microscope. Images were acquired using Volocity 3D Image Analysis Software (PerkinElmer, Waltham, Massachusetts, USA).

REFERENCES

1. Mimuro, H. et al. Grb2 is a key mediator of helicobacter pylori CagA protein activities. Mol Cell **10**, 745–755 (2002).

SUPPLEMENTAL DATASET

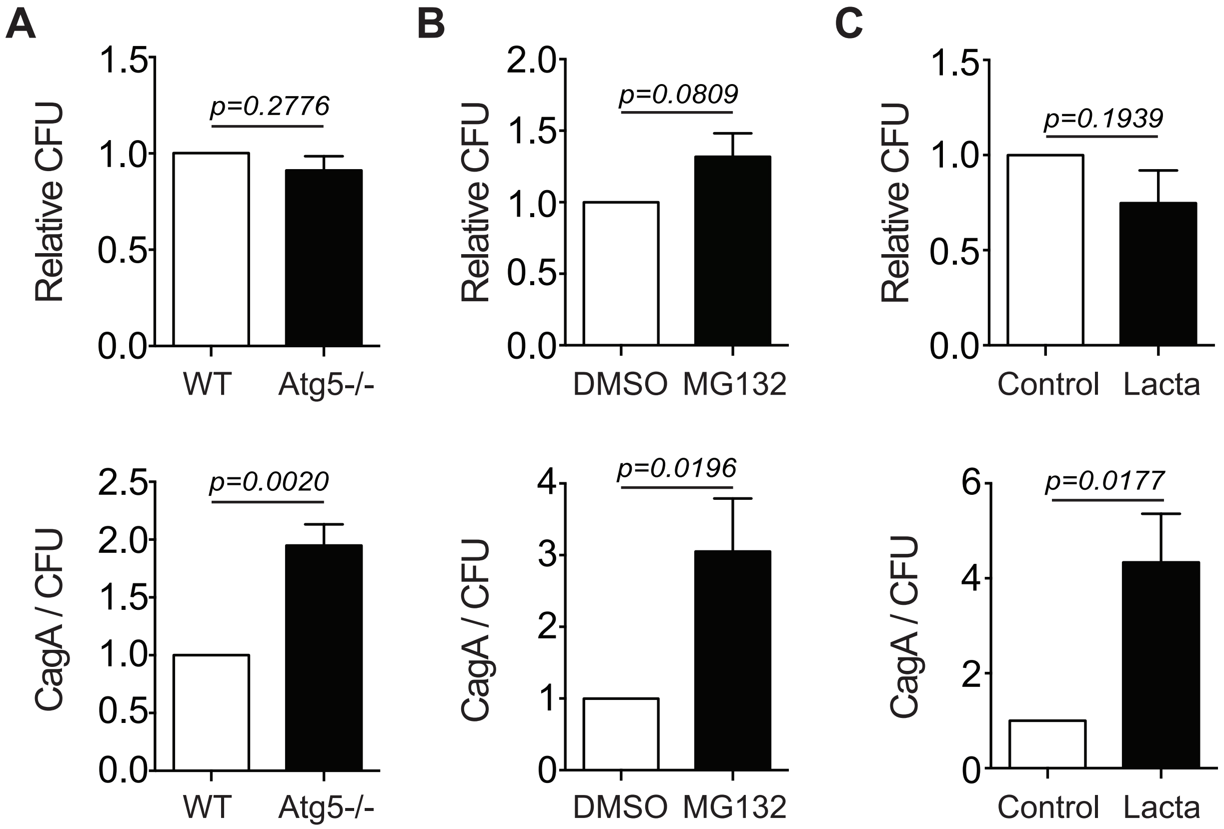

Figure S1. CagA accumulation is independent of bacterial survival. Bacterial viability assays were performed in parallel to experiments in Fig. 1 in order to calculate CFU. **(A)** WT and Atg5-/- MEFs were infected with a CagA+ *vacA-* isogenic mutant strain (MOI 100) for 8 hours using a gentamycin protection assay. Top graph shows the average CFU relative to WT and the bottom graph shows CagA protein level normalized to β-actin and CFU from each corresponding experiment (mean + SEM; n=4). **(B)** AGS cells were infected with a CagA+ *vacA-* isogenic mutant strain (MOI 50) for 19 hours using a gentamycin protection assay and treated with 5 μM MG132 or DMSO control for 14 hours. Top graph shows the average CFU relative to vehicle control and the bottom graph shows CagA protein level normalized to β-actin and CFU from each corresponding experiment (mean + SEM; n=6). **(C)** AGS cells were infected with a CagA+ *vacA-* isogenic mutant strain (MOI 50) for 24 hours using a gentamycin protection assay and treated with 10 μM Lactacystin or control for 19 hours. Top graph shows the average CFU relative to vehicle control and the bottom graph shows CagA protein level normalized to β-actin and CFU from each corresponding experiment (mean + SEM; n=4). Statistical analysis was performed using Student's t-test.

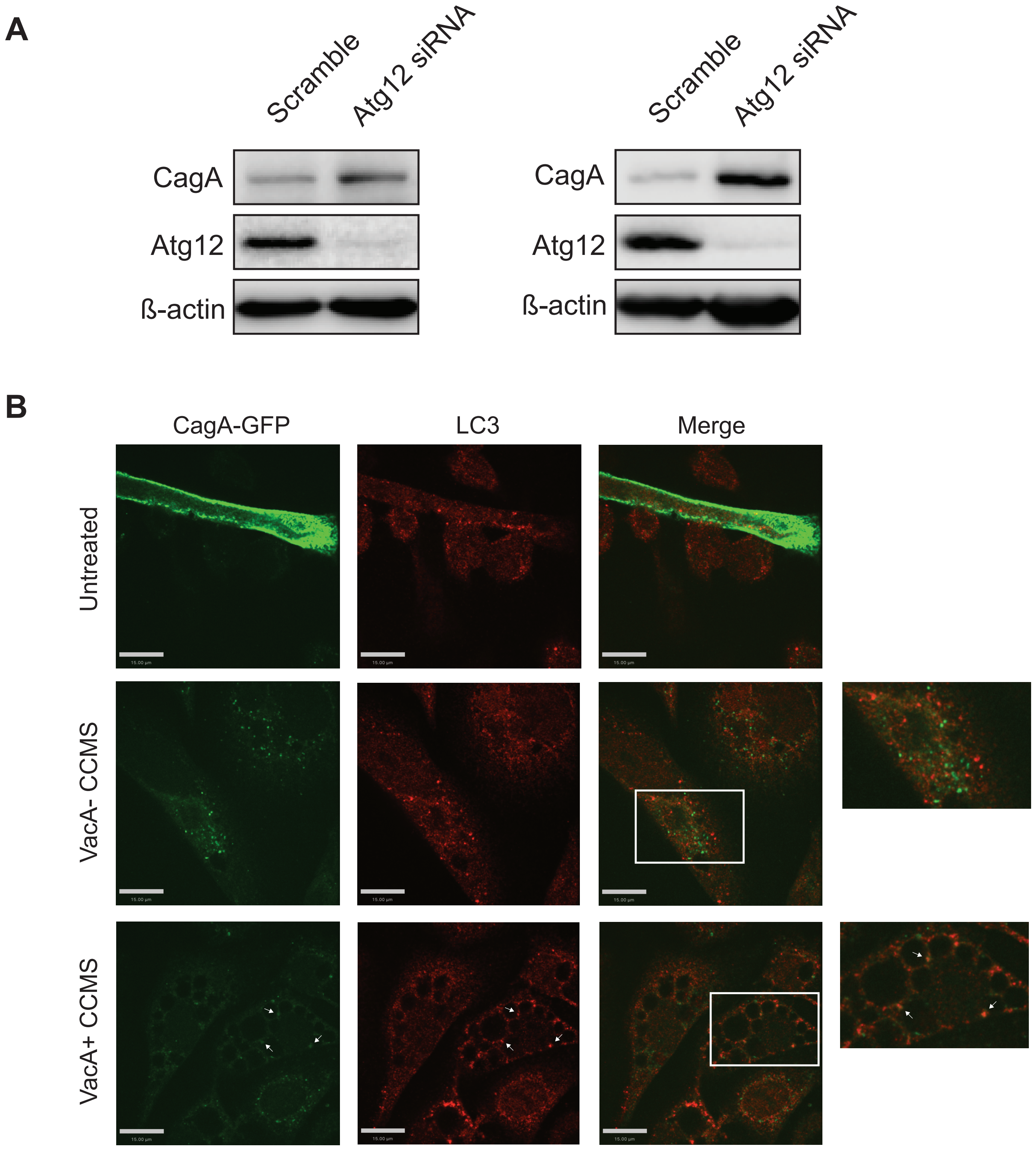

Figure S2. Inhibition of autophagy promotes CagA accumulation. **(A)** AGS cells were transfected with scramble or Atg12 siRNA and infected with a CagA+ *vacA*- isogenic mutant strain for 24 hours using a gentamycin protection assay. Knockdown was assessed by measuring Atg12 protein levels through Western blotting. CagA protein levels were measured by Western blotting using β-actin as loading control (n=2). **(B)** AGS cells were transfected with CagA-GFP and treated with VacA+ or VacA- CCMS or left untreated for 24 hours and subsequently stained for LC3. Inset shows colocalization of CagA and LC3 with arrows pointing to puncta. Scale bars are 15 μm.

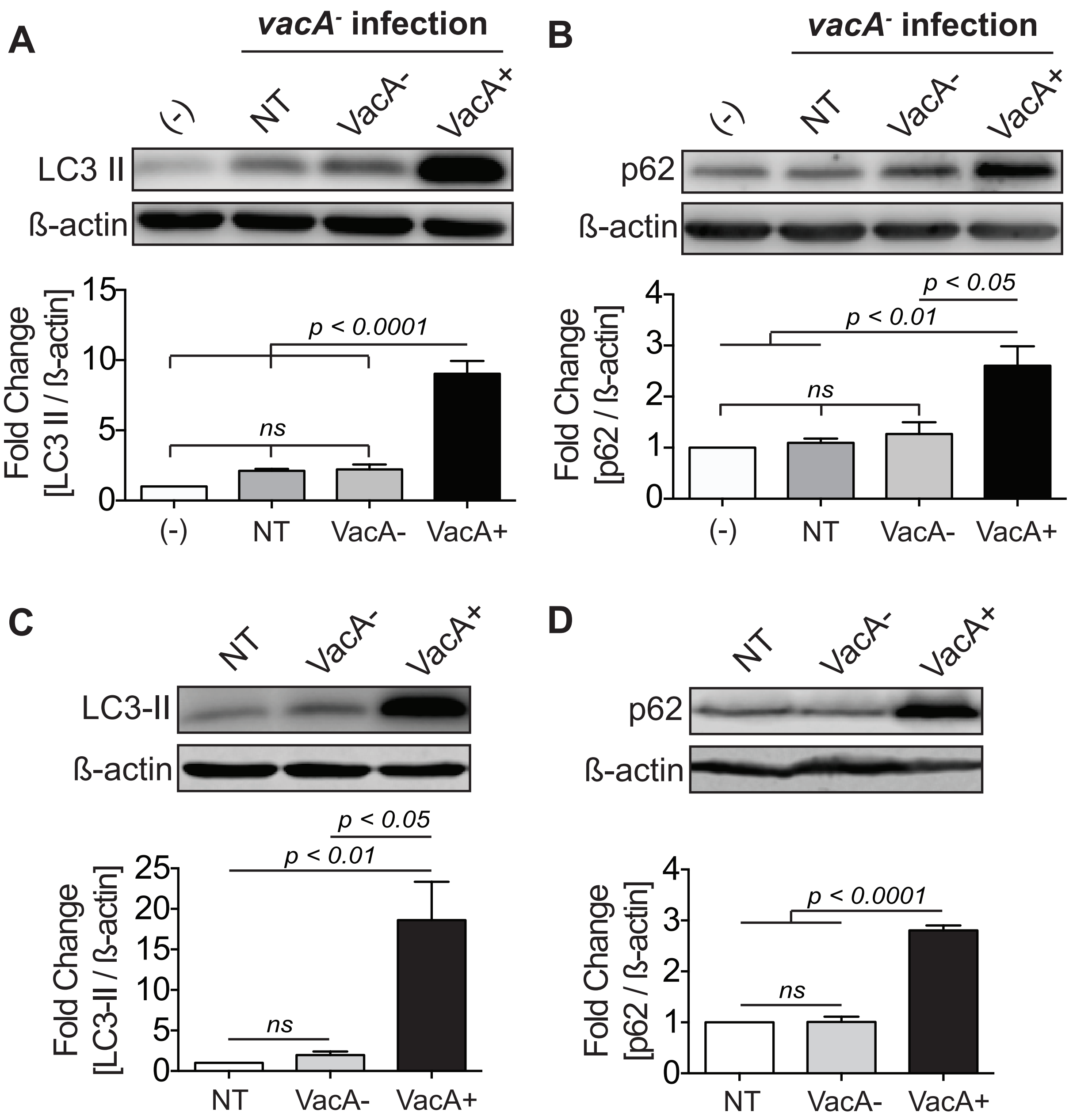

Figure S3. VacA treatment disrupts autophagic degradation. (A-B) AGS cells were infected with a CagA+ *vacA-* isogenic mutant strain and co-cultured in the presence or absence of VacA- or VacA+ CCMS for 24 hours using a gentamycin protection assay. NT denotes infection with a CagA+ *vacA-* isogenic mutant strain alone and (-) denotes uninfected control cells. **(A)** LC3-II was measured by Western blotting using β-actin as a loading control. Graph shows fold change of LC3-II normalized to β-actin relative to uninfected control cells (mean + SEM; n=4). **(B)** p62 was measured by Western blotting using β-actin as a loading control. Graph shows fold change of p62 normalized to β-actin relative to uninfected control cells (mean + SEM; n=3). **(C-D)** Ub-G76V-GFP HeLa cells were treated with VacA+ or VacA- CCMS for 32 hours. NT denotes untreated control cells. **(C)** LC3-II was measured by Western blotting using β-actin as a loading control. Graph shows fold change of LC3-II normalized to β-actin relative to NT (mean + SEM; n=3). **(D)** p62 was measured by Western blotting using β-actin as a loading control. Graph shows fold change of p62 normalized to β-actin relative to NT (mean + SEM; n=3). Statistical analysis was performed using ANOVA with Tukey's post-hoc test.

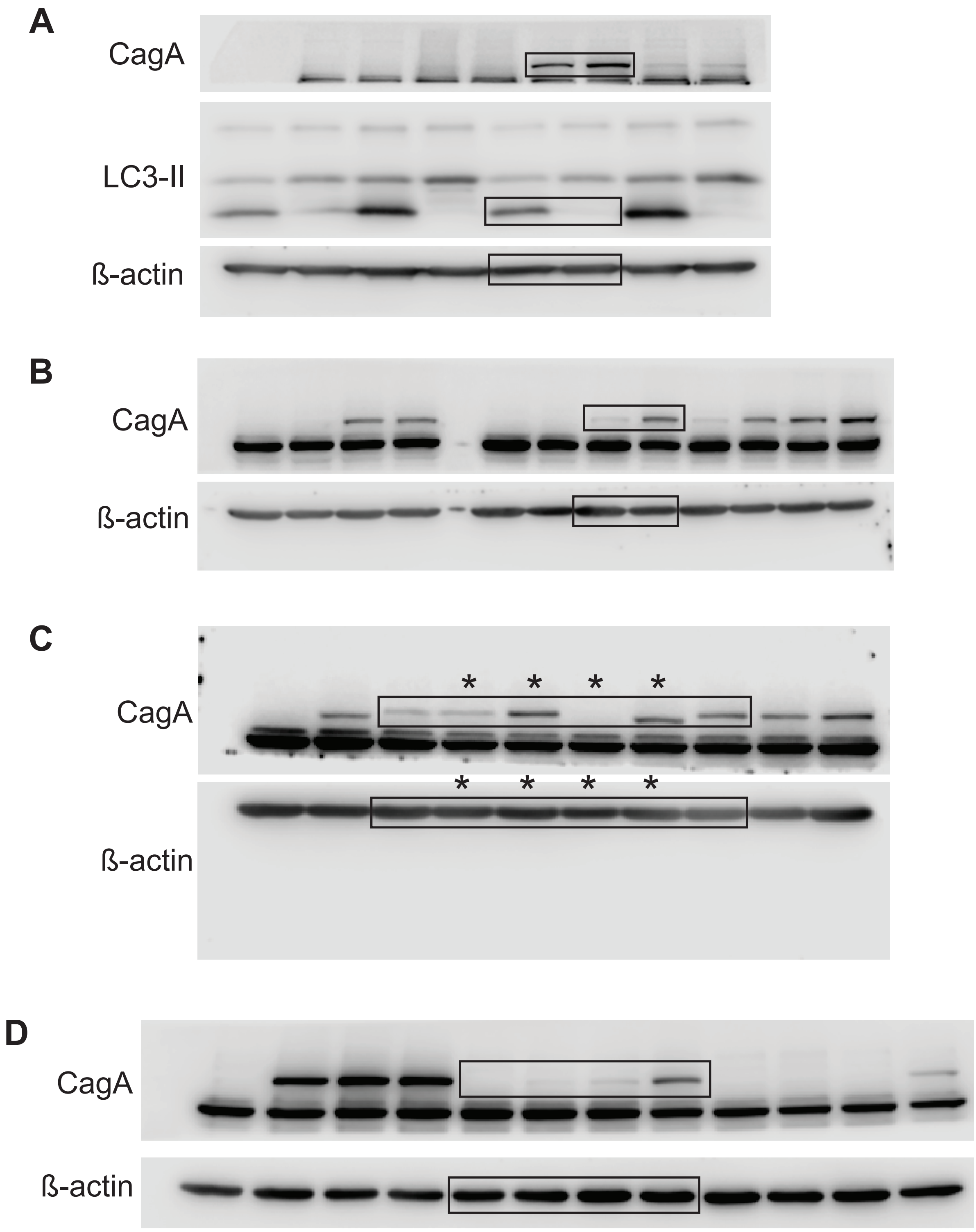

Figure S4. Original immunoblots used to crop the gel bands for **(A)** Figure 1A, **(B)** Figure 1B, **(C)** Figure 1C and **(D)** Figure 2A. * indicates the lanes removed to splice together the adjacent regions.

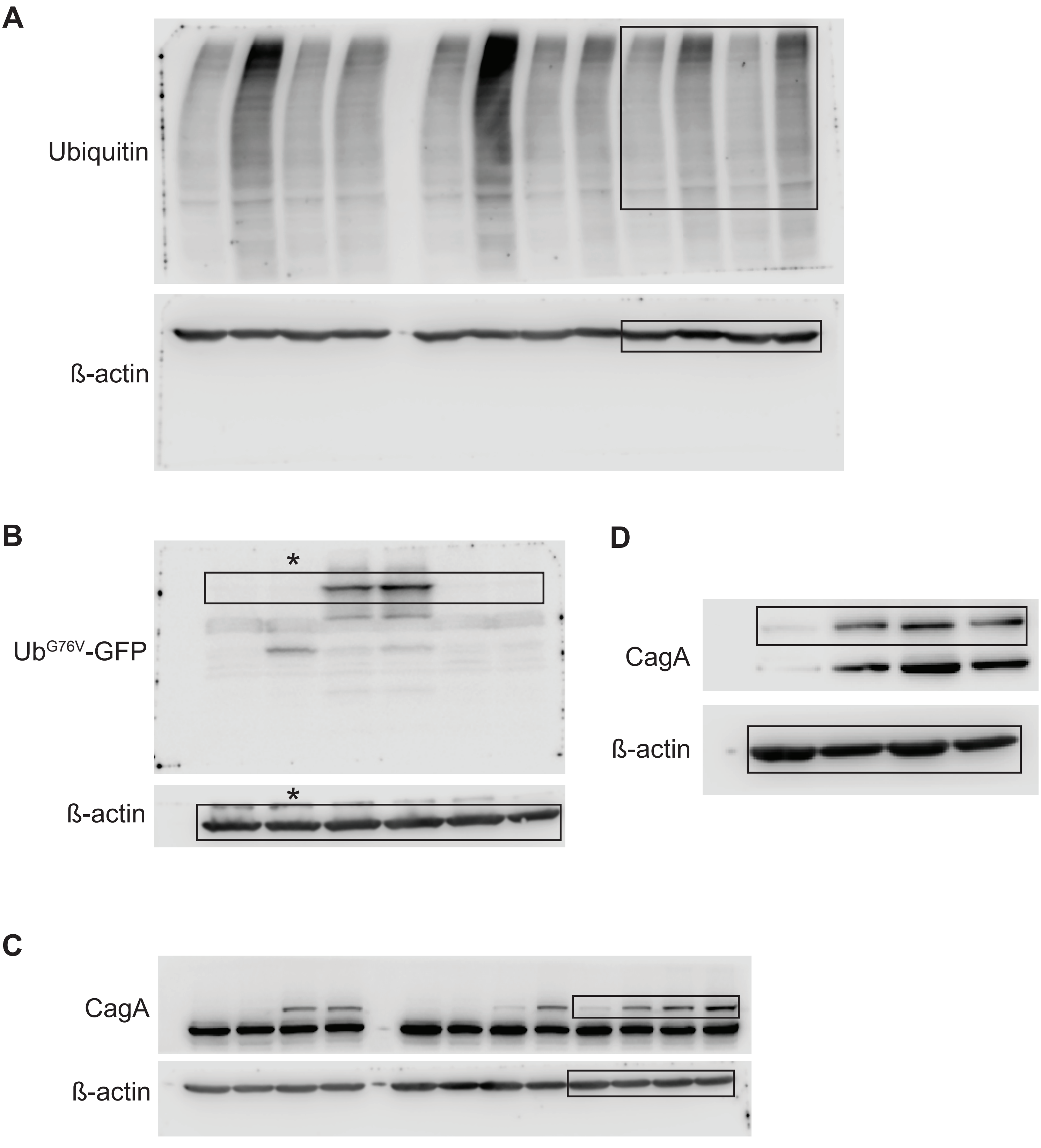

Figure S5. Original immunoblots used to crop the gel bands for **(A)** Figure 3A, **(B)** Figure 3B * indicates the lanes removed to splice together the adjacent regions, **(C)** Figure 4A and **(D)** Figure 4B.

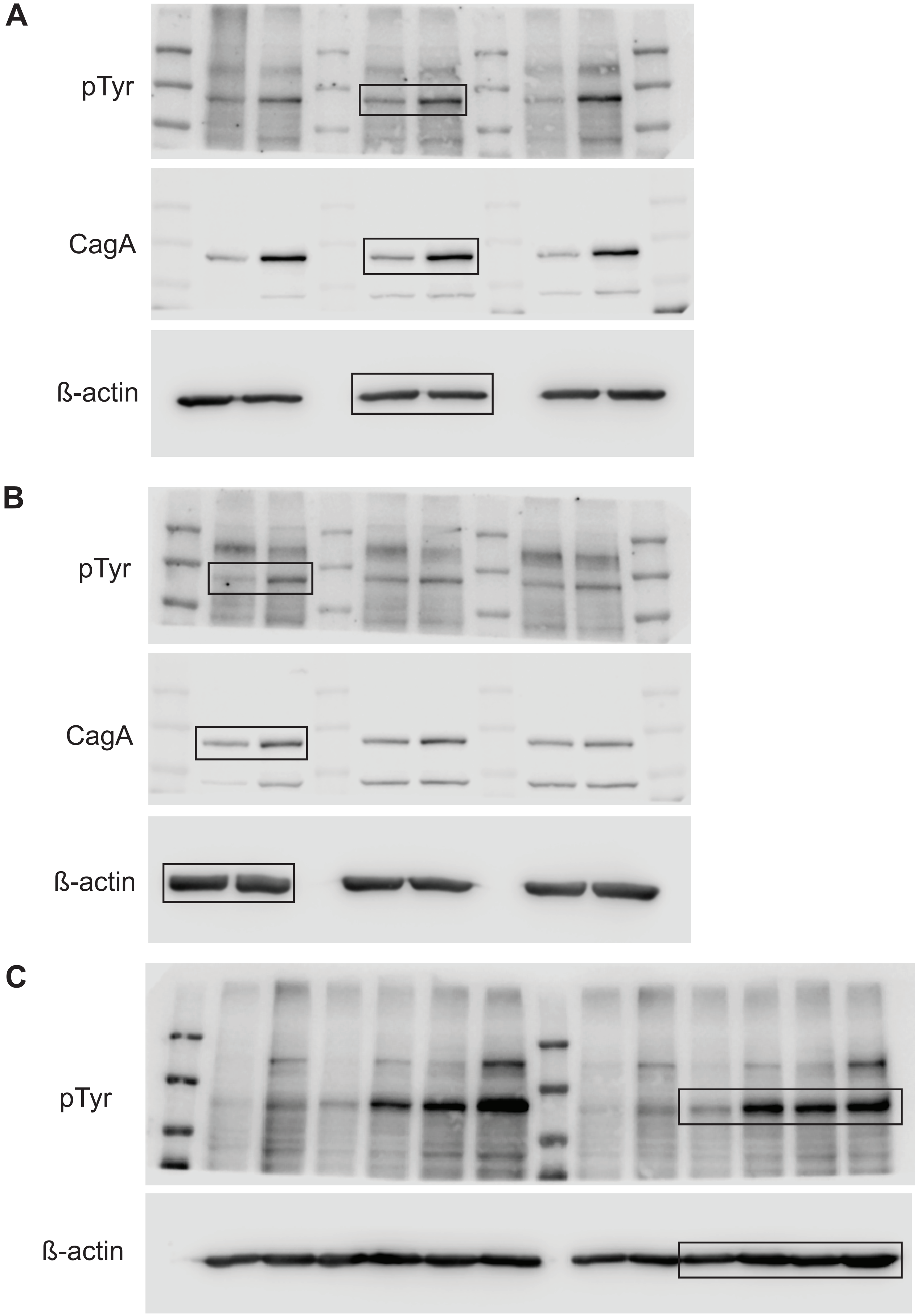

Figure S6. Original immunoblots used to crop the gel bands for **(A)** Figure 5A, **(B)** Figure 5B and **(C)** Figure 5C.
